# A Chromo-Fluorogenic Naphthoquinolinedione-Based Probe for Dual Detection of Cu^2+^ and Its Use for Various Water Samples

**DOI:** 10.3390/molecules27030785

**Published:** 2022-01-25

**Authors:** Ashwani Kumar, Subodh Kumar, Pil Seok Chae

**Affiliations:** 1 Department of Bionano Engineering, Hanyang University, Ansan 15588, Korea; 2 Department of Chemistry, UGC Center for Advanced Studies, Guru Nanak Dev University, Amritsar 143005, India; subodh.chem@gndu.ac.in

**Keywords:** fluorescence, bis(triazole)-conjugated naphthoquinolinedione, Cu^2+^ sensing/bio-imaging, colorimetric/ratio-metric

## Abstract

The presence of an abnormal amount of Cu^2+^ in the human body causes various health issues. In the current study, we synthesized a new naphthoquinolinedione-based probe (probe **1**) to monitor Cu^2+^ in different water systems, such as tap water, lakes, and drain water. Two triazole units were introduced into the probe via a click reaction to increase the binding affinity to a metal ion. In day-light, probe **1** dissolved in a mixed solvent system (HEPES: EtOH = 1:4) showed a vivid color change from light greenish-yellow to pink in the presence of only Cu^2+^ among various metal ions. In addition, the green luminescence and fluorescence emission of the probe were effectively bleached out immediately after Cu^2+^ addition. The limit of detection (LOD) of the probe was 0.5 µM when a ratio-metric method was used for metal ion detection. The fluorescence titration data of the probe with Cu^2+^ showed a calculated LOD of 41.5 pM. Hence, probe **1** possesses the following dual response toward Cu^2+^ detection: color change and fluorescence quenching. Probe **1** was also useful for detecting Cu^2+^ spiked in tap/lake water as well as the cytoplasm of live HeLa cells. The current system was investigated using ultraviolet-visible and fluorescence spectroscopy as well as density functional theory calculations (DFT).

## 1. Introduction

Metal ions play essential roles in a variety of biochemical processes in the human body, such as osmotic pressure and pH regulation, signaling transduction, and metabolism [1,2,3,4,5,6]. The concentrations of these metal ions need to be regulated for their individual functions. Abnormal concentrations can result in the malfunctioning or non-functioning of different organs that could eventually have detrimental effects on human health, such as physical disorders and chronic/acute diseases [7,8,9,10,11,12]. Therefore, selective and sensitive detection is necessary for individual metal ions that need to be monitored and regulated, especially in drinking water (e.g., tap/lake water) [13,14,15,16,17,18]. Heavy metal ions can be toxic to mammals but also play important physiological roles [19,20,21,22,23,24]. Cu^2+^ has the third largest concentration of all heavy metal ions in the human body. This metal ion helps to sustain immune function, nerves, bones, blood vessels, and metabolism. In addition, Cu^2+^ serves a critical function in iron absorption [25,26]. However, excessive Cu^2+^ exposure, even for a limited period of time, can lead to serious health concerns such as liver/kidney problems, gastrointestinal (GI) dysfunction, and many serious neurodegenerative diseases (e.g., Menkes disease) [27,28,29,30,31,32,33,34,35,36]. As Cu^2+^ is extensively used in electronics, transport, and construction and many of its salts are being used as supplementary components in medicine and agriculture, this metal ion readily contaminates drinking water, posing a potential threat to human health [37,38,39,40]. The limit for Cu^2+^ concentration in drinking water is 1.3 ppm (~20 µM), as set by the U.S. Environmental Protection Agency [41]. Due to health concerns associated with over-exposure to Cu^2+^, many scientists have made efforts toward a convenient, reliable, selective, and efficient sensor for effective risk assessment of this metal ion in environmental and biological samples [42,43,44,45,46,47,48,49,50,51].

A variety of analytical techniques have been developed to precisely detect metal ions in biological and environmental samples. The numerous types include ion-selective membrane electrodes, atomic fluorescence spectrometry, X-ray fluorescence spectrometry, atomic absorption spectrometry, voltammetry, and inductively-coupled plasma mass spectroscopy [52,53,54,55,56,57,58,59,60,61]. These techniques have a number of limitations, such as the existence of basic interferences, time-consuming procedures, high cost, and need for highly skilled technicians to perform experiments. In contrast, chromo-fluorogenic sensors are advantageous in terms of easy operation, simplicity, affordability, on-site quick monitoring, and naked-eye detection. In addition, the highly sensitive and selective nature of chromo-fluorogenic sensors has inspired the scientific community to use an optical approach for detection of various analytes [62,63,64,65,66,67,68,69]. Many scientists have made efforts to develop new probes suitable for this application [70,71,72,73,74,75,76,77]. Thus, development of a new hetero-aromatic platform with chromo-fluorogenic properties is important for use in supramolecular chemistry and other interdisciplinary sciences. Keeping these ideas in mind, we synthesized a novel probe using an amino-functionalized naphthoquinolinedione platform (probe **1**) for selective and distinctive detection of Cu^2+^ in an EtOH-based water solution. The probe introduced in this study is capable of selectively recognizing Cu^2+^ by absorption and luminescence/fluorescence change. The color of probe **1** changed from light-yellow to pink in aqueous solutions upon Cu^2+^ binding. The intense green emission of the probe was dramatically quenched in the presence of Cu^2+^, giving a very low limit of detection (LOD) of 41.5 pM (calculated). In addition, we successfully applied this sensing system for metal ion detection in real (tap/lake water) and bio-samples (HeLa cells).

## 2. Results and Discussion

### 2.1. Synthesis and Characterization of Probe ***1***

**Scheme 1:**
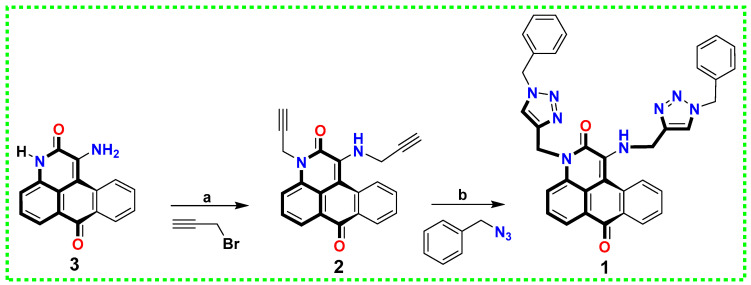
Synthetic scheme of probe **1**; (a) K_2_CO_3_, DMSO, RT, 24 h; (b) Na(ascorbate), CuSO_4_⋅5H_2_O, DCM: EtOH (1:1), 3 h. Heteroatoms were color-coded for clarity: nitrogen (blue), oxygen (red).

Probe **1** was synthesized via a click reaction between benzyl azide and a dipropargylated derivative (**2**) prepared from amino-functionalized naphthoquinolinedione (compound **3**) by simple alkylation (Scheme 1). The chemical structures of probe **1** and compounds **2** and **3** were characterized by ^1^H and ^13^C nuclear magnetic resonance (NMR) spectroscopy. The amine group-bearing naphthoquinolinedione derivative (compound **3**) was reacted with propargyl bromide in dimethyl sulfoxide (DMSO) using K_2_CO_3_ (base) and tetrabutylammonium bisulfate (TBAHSO_4_; catalyst) to produce compound **2** (70%). The formation of compound **2** was verified by loss of amidic NH of compound **3** at 12.35 ppm and the appearance of two alkyne peaks (≡C-H) as singlets at 2.26 and 2.36 ppm. Two doublets at 3.85 and 5.26 ppm corresponding to the methylene protons (CH_2_) of the propargylic groups further support the formation of compound 2 with two propargylic groups. A click reaction of benzyl azide with di-alkyne-functionalized compound **2** gave probe **1** in 74% yield. The ^1^H NMR spectrum showed loss of the alkyne CH peaks and appearance of one doublet at 4.31 ppm, corresponding to the α-CH_2_ of the amine NH group. The amine NH peak appeared as a triplet at 6.59 ppm, while the three singlets at 5.40, 5.44, and 5.68 ppm correspond to two benzylic protons (CH_2_) and amide nitrogen-linked methylene protons (CH_2_), respectively.

### 2.2. Spectroscopic Characterization and Sensing Ability of Probe ***1***

UV-vis absorption of probe **1** was performed in ethanol in the presence of various metal ions including monovalent (Na^+^, Ag^+^, and K^+^), divalent (Mg^2+^, Ca^2+^, Ba^2+^, Fe^2+^, Co^2+^, Zn^2+^, Cd^2+^, Pb^2+^, Cu^2+^, Ni^2+^, and Hg^2+^), and trivalent ions (Cr^3+^, Fe^3+^, Ga^3+^, Al^3+^, and Ru^3+^). Probe **1** (10 µM, EtOH) showed a strong band at 438 nm originating from *n →* π* transition, and additions of various metal ions induced little change in most cases (Figure S1a). The additions of Ag^+^, Co^2+^, and Ni^2+^ resulted in a decrease in the intensity of the original absorption band and appearance of a new band in the range of 500 to 550 nm, but the changes in absorbance were small. Upon addition of Cu^2+^, the probe solution showed a large change in the UV-vis spectrum; a large absorption band appeared at 505 nm, along with almost complete loss of the original band at 438 nm. A similar behavior was observed for the fluorescence spectra of the probe (Figure S1b). The probe solution gave a strong fluorescence emission at 524 nm, which was substantially decreased (~15% quenching) upon addition of Ag^+^, Co^2+^, or Ni^2+^. By contrast, Cu^2+^ addition resulted in 90% quenching of the fluorescence emission of the probe.

**Figure 1:**
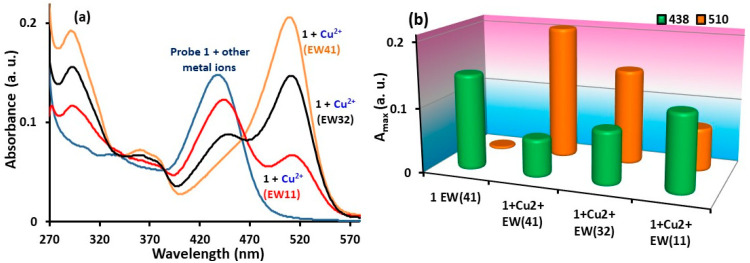
Effect of water content on Cu^2+^ sensing of probe **1** (10 µM, EtOH). (**a**) UV-vis spectra of probe **1** with increasing water content in EtOH from 0% (i.e., EtOH only) to 50% water and (**b**) associated bar diagram of the absorption maximum value before (***A***_438_) and after addition of Cu^2+^ (***A***_510_). ‘E’ and ‘W’ represent ethanol and water, respectively, and the numbers indicate the ratios of ethanol and water.

To find an optimal condition for selective metal ion sensing, probe **1** was applied for Cu^2+^ sensing in different solvent systems. When Cu^2+^ was added to a 20% aqueous solution of ethanol containing probe **1**, designated EW (41), no metal ions except Cu^2+^ produced a meaningful change in the UV-vis spectrum of the probe (Figure 1a). The addition of Cu^2+^ led to a significant reduction in absorbance at 438 nm and appearance of a new band at 510 nm, indicating complex formation between probe **1** and the metal ion. When water concentration was increased to 40% (EW32) or 50% (EW11), Cu^2+^-induced spectral changes became less prominent than those with the 20% solution (Figure 1). A similar trend was observed when the fluorescence study of probe **1** was carried out in different solvent conditions (Figure S2). The probe showed a dramatic change (>85% quenching) in fluorescence intensity at 535 nm (***I***_535_) upon addition of Cu^2+^ when dissolved in 20% aqueous solution (EW41). Uses of 50% and 40% water in EtOH produced ~30% and ~50% fluorescence reductions, respectively, upon Cu^2+^ addition. Thus, we used a 20% aqueous EtOH solution as a medium for Cu^2+^ detection in this study. Of note, the other metal ions showed minimal changes in the fluorescence spectrum of probe **1**.

**Figure 2:**
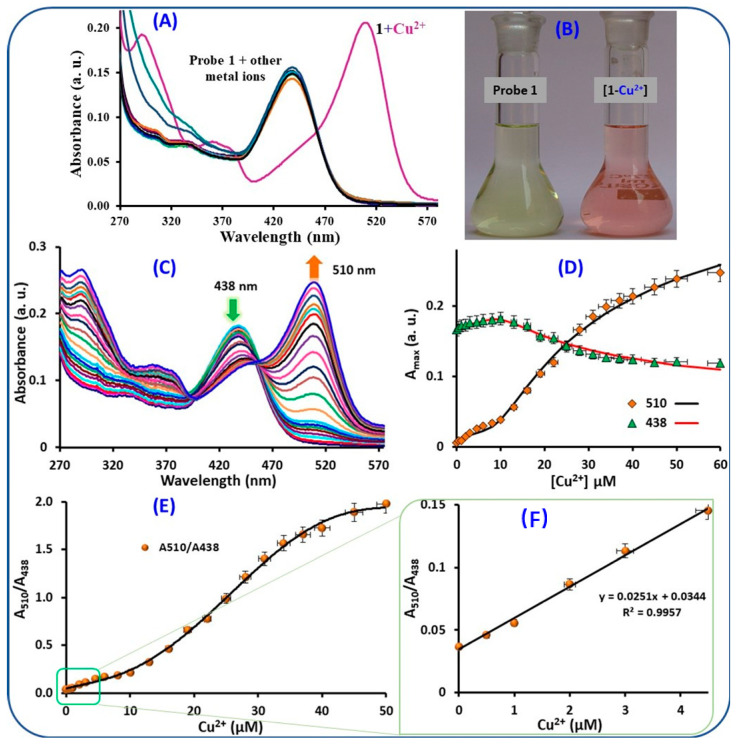
(**A**) UV-visible spectra of probe **1** (10 µM, HEPES (pH 7.4): EtOH = 1:4) upon additions of various metal ions. (**B**) Color change of the probe solution in daylight upon addition of Cu^2+^. (**C**) UV-visible titration of probe **1** with Cu^2+^. (**D**) Fitting of titration data using absorption values at 438 and 510 nm (***A***_438_ and ***A***_510_). (**E**) Change in absorbance ratio (***A***_510_/***A***_438_) as a function of [Cu^2+^]. (**F**) Expanded version of (E) focusing on a low [Cu^2+^] range (0–4.5 µM). Points and curves represent experimental values and curve fits, respectively.

Upon addition of Cu^2+^ to probe **1** (10 µM, HEPES (pH 7.4): EtOH = 1:4), a vivid color change from light greenish-yellow to pink was observed, consistent with the spectroscopic results obtained under the same conditions (Figure 2A,B). Based on this result, we carried out absorbance titration experiments of the probe with Cu^2+^. Over the course of the titration, the absorbance of probe **1** at 438 nm decreased gradually with increasing concentration of Cu^2+^, along with a concomitant increase in absorbance at 510 nm. Accordingly, we found two isosbestic points at 393 and 451 nm (Figure 2C,D). The spectral saturation of the probe was detected at the addition of ~40 µM Cu^2+^. Nonlinear regression analysis of this titration data suggests the formation of 1:1 complex with a high association constant (*K_a_* = 7.04 × 10^6^ M^−1^). A ratio-metric approach using ratio of two absorption values (***A***_438_ and ***A***_510_) allowed us to estimate Cu^2+^ in the range of 0.5 to 50 µM, with an LOD of 0.5 µM (Figure 2E,F). The additions of different metal ions to probe **1** solution containing Cu^2+^ only slightly altered the absorption spectrum, indicating that these metal ions failed to interfere with Cu^2+^ detection by the probe (Figure S3A). 

**Figure 3:**
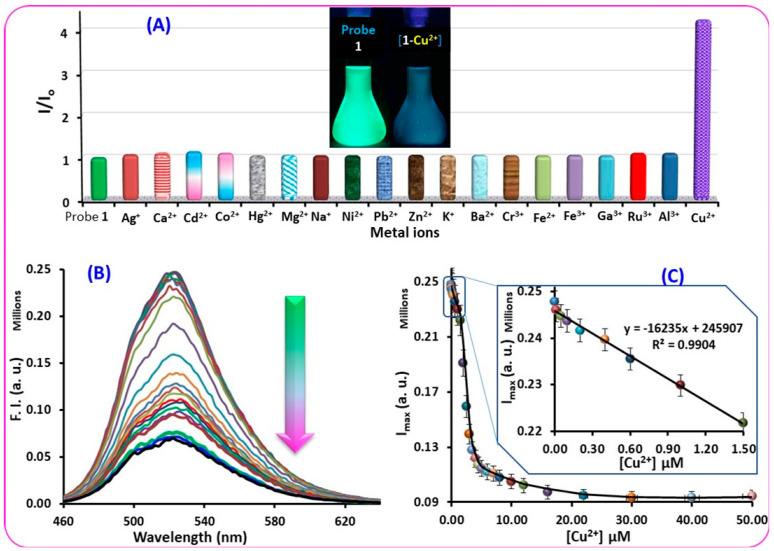
(**A**) Bar diagram of fluorescence intensity ratio (***I***_o_/***I***) of probe **1** (10 µM, HEPES (pH 7.4): EtOH = 1:4) upon additions of various metal ions (200 µM each). ***I***_o_ and ***I*** are the fluorescence intensities of probe **1** measured at 524 nm before and after additions of the individual metal ions, respectively. The inset shows a luminescence change of the probe solution upon Cu^2+^ addition under irradiation at 365 nm. (**B**) Fluorescence titration of the probe with Cu^2+^ and (**C**) associated data and fit curve using a fluorescence emission intensity at 524 nm (***I***_524_). The inset shows a linear relationship between [Cu^2+^] and ***I***_max_ in a low [Cu^2+^] range (0–1.5 µM); *λ_ex_* = 435 nm.

Under excitation at 435 nm, probe **1** (10 µM, HEPES (pH 7.4): EtOH = 1:4) exhibited a strong fluorescence emission intensity at 524 nm and a high quantum yield (*Φ* = 0.33). Among the various metal ions, the probe responded only to the addition of Cu^2+^ by quenching of green luminescence and fluorescence intensity of probe **1** (Figure 3A). The titration of the probe with Cu^2+^ produced a gradual decrease in the emission intensity at 524 nm (***I***_524_) with increasing Cu^2+^ concentration (Figure 3B). According to the fluorescence titration study, the probe could detect Cu^2+^ as low as 0.01 µM (inset of Figure 3C) and the calculated LOD was 41.5 pM (Figure S4). This LOD value of probe **1** is comparable to or better than the values obtained from previously reported probes (Table S1). A nonlinear regression analysis of the fluorescence titration data indicates the formation of a 1:1 complex between probe **1** and Cu^2+^, with a large association constant (*K_a_* = 7.06 × 10^6^ M^−1^) (Figure 3C), which is consistent with the result of the UV-vis titration study. This was further confirmed by the mass data of the [**1**-Cu^2+^]-complex giving a sharp peak at 667.1627, close to a theoretical value of 667.1620 (see ESI).

### 2.3. Density Functional Theory (DFT) Calculations for Probe ***1*** and Its Cu^2+^ Complex

**Figure 4:**
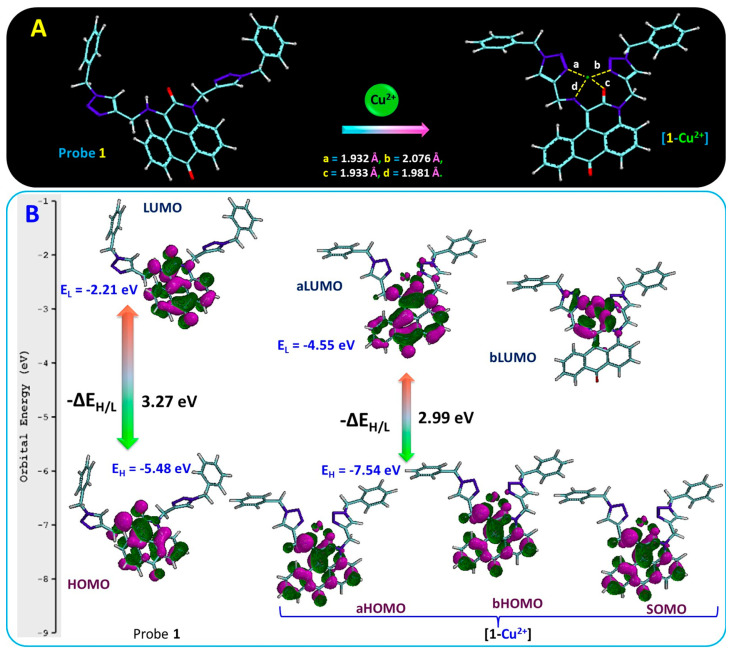
(**A**) Energy-minimized structures of probe **1** and [**1**-Cu^2+^]-complex (ball and wire model) and (**B**) electronic distributions and energies of their frontier molecular orbitals (FMOs; HOMOs and LUMOs) obtained from DFT calculations at the B3LYP/6-31G* level. Atoms are color-coded: N = blue, O = red, C = cyan, and H = grey.

**Scheme 2:**
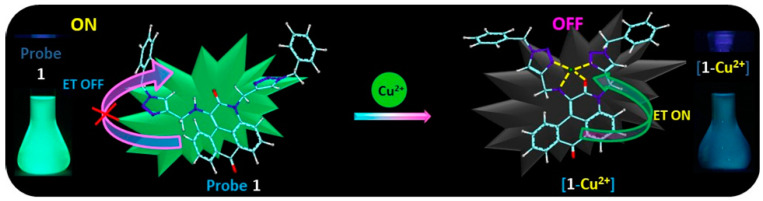
Schematic representation of a plausible mechanism responsible for fluorescence quenching of probe **1** upon addition of Cu^2+^. Metal ion binding to the probe leads to an electron transfer from the naphthoquinolinedione ring to the metal binding site, resulting in fluorescence quenching in the [**1**-Cu^2+^] complex.

To investigate the molecular interactions of probe **1** with Cu^2+^, we obtained the optimized geometries of probe **1** and of its Cu^2+^-complex via DFT at the B3LYP/6-31G* level (Figure 4 and Figure S5). In addition, these calculations provide information about the energy levels and electron distributions of the frontier orbitals (i.e., highest occupied molecular orbital [HOMO] and lowest unoccupied molecular orbital [LUMO]). Based on the results of UV-vis and fluorescence titration studies, we used a 1:1 complex between probe **1** and the metal ion as a model for the calculations. The calculations indicate the coordination of Cu^2+^ to a binding site of the probe consisting of four atoms (i.e., amine N, two nitrogen atoms of two triazole rings and carbonyl O), resulting in metal ion complex formation with a tetrahedral geometry (Figure 4A and Figure S5). Upon complex formation, the HOMO and LUMO energy gap (Δ*E_H/L_*) was reduced from 3.27 to 2.99 eV (Figure 4B). This reduced energy gap is consistent with the red-shift of the absorption peak from 438 to 510 nm upon Cu^2+^ binding of the probe. The HOMO electron of probe **1** is mainly distributed on the naphthoquinolinedione platform. Similar electronic distributions were obtained for the aHOMO, bHOMO, and SOMO of probe **1** complexed with Cu^2+^. However, as indicated by the bLUMO of the complex, the electron density of LUMO is transferred from the naphthoquinolinedione unit to the metal-binding site upon metal ion complexation (Figure 4B). This electron transfer is likely responsible for the effective quenching of the green fluorescence emission of probe **1** upon Cu^2+^ binding (Scheme 2). However, it is also possible that the paramagnetic character of Cu^2+^ is partly responsible for the fluorescence quenching, as reported in literatures [71]. 

**Figure 5:**
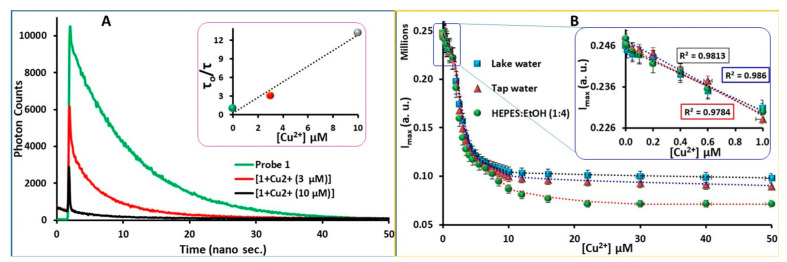
(**A**) Fluorescence decay profiles of probe **1** (10 µM) with increasing concentration of Cu^2+^ from 0 to 0.3 (3 µM) to 1.0 equivalent (10 µM). The probe was dissolved in a solvent system (HEPES (pH = 7.4): EtOH = 4:1). (**B**) Plot of the fluorescence intensity of probe **1** (10 μM) as a function of [Cu^2+^] in three different media (HEPES, tap, and lake waters). The data were obtained from titration of the probe with Cu^2+^ and fluorescence intensity measured at 524 nm (***I***_524_). The inset in (**A**) shows changes in the ratio of excited state life-time (***τ***_o_/***τ***), where ***τ***_o_ and ***τ*** represent excited state life-times of the probe in the absence and presence of Cu^2+^, respectively. The inset in (**B**) shows a linear relationship between ***I***_524_ and [Cu^2+^] in a range of low metal ion concentration. The points and curves are the experimental values and curve fits, respectively.

Static and dynamic processes are two main pathways leading to fluorescence quenching. To investigate which process is dominantly involved, we obtained a Stern–Volmer plot (Figure S6) and measured an excited state life-time (***τ***) of probe **1** with increasing amount of Cu^2+^ (Figure 5A). In the Stern–Volmer plot, the fluorescence intensity of the probe was linearly decreased with increasing [Cu^2+^], suggestive of occurrence of dynamic quenching process. In the measurements of excited state life-time, the probe gave a reduced life-time from 9.7 to 3.2 ns when 0.3 equivalent (3 µM) Cu^2+^ was added to the probe (inset of Figure 5). The excited state life-time of the probe was further decreased to 0.7 ns with increasing the amount of Cu^2+^ to 1.0 equivalent (10 µM). Based on the linearity of Stern–Volmer plot and the reduced excited state life-time, a dynamic process is likely dominant over a static one for the fluorescence quenching observed for the [probe **1**-Cu^2+^] complex. Of note, a static process can also contribute the observed fluorescent quenching to some extent.

### 2.4. Practical Application of Probe ***1***

**Figure 6:**
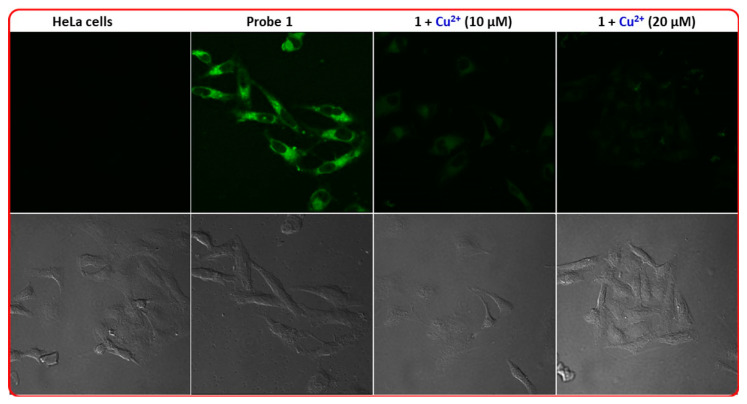
Confocal microscopic images of HeLa cells in the absence and presence of probe **1** and/or Cu^2+^. HeLa cells were preloaded with probe **1** (10 µM) for 30 min and then treated with the designated amount of Cu^2+^ (0, 10, or 20 µM), followed by acquisition of fluorescence images under excitation at 488 nm. The 1st panel (**top**) was obtained using a green channel, while the 2nd panel (**bottom**) corresponds to bright-field images of the cells.

To explore utility in practical applications, we first investigated the ability of the probe to detect Cu^2+^ in tap or lake water. Fluorescence titrations of the probe were carried out using Cu^2+^-spiked samples prepared from tap/lake water and EtOH. Probe **1** dissolved in mixed solvent systems [water: EtOH = 1:4] gave LODs of 10 nM in both cases, which is the same value as with the HEPES buffer system [i.e., HEPES: EtOH = 1:4]. This result indicates that the probe has potential for on-site monitoring of Cu^2+^ in drinking water (Figure 5B). Probe **1** was further investigated in the context of its ability to detect Cu^2+^ in live HeLa cells. The experiment started with incubation of the HeLa cells with probe **1** for 30 min. Under irradiation at 488 nm, the cells with no probe displayed negligible emission, but 30 min incubation of the cells with probe **1** at 10 µM resulted in a high fluorescence emission in the cytoplasm (Figure 6 and Figure S7). The high fluorescence emission in the cytoplasm rather than the nucleus indicates that the probe penetrated the plasma membranes but did not pass through the nuclear membranes. When the HeLa cells containing probe **1** were treated with 10 µM Cu^2+^, the green emission was reduced substantially. After further addition of 10 µM Cu^2+^ (20 µM in total), the green emission of the probe was lost, presumably due to complexation with the metal ion (Figure 6). Bright-field images indicate that these cells were viable throughout the experiment. Therefore, intracellular Cu^2+^ can be detected in a non-toxic manner using probe **1**, which is important for bio-applications of this probe.

## 3. Materials and Methods

### 3.1. General Chemicals and Materials

All reagents and solvents used in the experiments, including propargyl bromide (80% toluene), all metal perchlorates, TBAHSO_4_, DMSO, potassium carbonate, and ethanol were purchased from Aldrich (analytical grade) and were used without further purification. 1-Amino-3*H*-naphtho[1,2,3-*de*]quinoline-2,7-dione (compound **3**) was synthesized according to a procedure reported previously [78]. All the fluorescence spectra were recorded on a ChronosBH fluorescence lifetime spectrometer. Ultraviolet-visible (UV-vis) spectra were recorded on Shimadzu UV-2450 and Shimadzu UV-2600PC spectrophotometers with a quartz cuvette. The cell holder was maintained at 25 °C. ^1^H and ^13^C NMR spectra of all new compounds were recorded on a Bruker 400 MHz spectrophotometer using CDCl_3_ as a solvent and tetramethylsilane (TMS) as an internal standard. NMR data were reported as chemical shifts in parts per million (*δ*), multiplicities (s = singlet, d = doublet, m = multiplet), coupling constants (Hz), integration, and interpretation. All spectrophotometric titration curves were fitted with gnu plot software.

### 3.2. Parameters and Conditions for Photo-Physical Studies of Probe ***1***

All metal ion-induced color changes and UV-vis and fluorescence spectra were obtained using ethanol solvent or a binary solvent (HEPES (pH 7.4): EtOH (1:4)). All absorption and fluorescence scans were saved as ACSII files and further processed in Excel^TM^ to produce the graphics shown. A stock solution of probe **1** was prepared at 1 mM in DMSO, and it was used for photo-physical studies following an appropriate dilution with ethanol or a binary solvent (HEPES (pH 7.4): EtOH (1:4)). Both UV-vis and fluorescence studies were performed using the probe at 10 µM. Association constants between the probe and Cu^2+^ were determined by fitting the absorption and fluorescence spectral data obtained from the titration experiment of probe **1** with Cu^2+^. The titration data were fitted with the global analysis program SPECFIT-32. For interference studies, the absorbance and fluorescence emission intensities were measured in a series of solutions (HEPES (pH 7.4): EtOH (1:4)) containing chemo-sensor **1** (10 µM), Cu^2+^ (2 equiv.), and each interfering metal ion (100 eq. or 1 mM). Time-resolved fluorescence (TRF) measurements were carried out using a confocal microscope spectrometer (MicroTime-200, Picoquant, Germany) with a 10× (UPlanXApo, Olympus) objective. The excited state life-time measurements were performed at the Korea Basic Science Institute (KBSI), Daegu Center, Korea. A single-mode pulsed diode laser (470 nm with a pulse width of ~30 ps and an average power of 0.07–1.0 μW in 5 MHz repetition rate) was used as an excitation source. A dichroic mirror (490 DCXR, AHF) and a single photon avalanche diode (PDM series, MPD) were used to collect emission from the samples. A time-correlated single-photon counting system (PicoHarp300, PicoQuant GmbH, Berlin, Germany) was used to count emission photons. Exponential fitting for the fluorescence decay data was performed using the Symphotime-64 software (Ver. 2.2). 

### 3.3. LOD Calculation of Probe ***1*** for Cu^2+^ Detection

The fluorescence titration data was used to calculate an LOD of the probe. The plot of the maximum fluorescence intensity of the probe (***I***_524_) as a function of [Cu^2+^] produced a linear curve with a slope (*K*). The fluorescence emission spectra of the probe were measured three times, and a standard deviation (*σ*) of the blank measurements was obtained. An LOD was calculated using the following equation [79]:

LOD = 3 × *σ*/*K*

### 3.4. Theoretical Calculations 

Energy-minimized structures of probe **1** and its Cu^2+^ complex were obtained via gradient-correlated density functional theory (DFT) calculations using Becke’s three-parameter exchange functional [80] and the Lee-Yang-Parr (B3LYP) exchange correlation function [81] with 6-31G* basis sets for C, H, N, and O. B3LYP/6-31G* calculations were employed for excited-state optimization. For simplicity, all calculations were carried out in a vacuum and solvent effects were not included in the calculations. All stationary points were verified as the minima via calculations from Hessian and harmonic frequency analyses [82,83].

### 3.5. Synthesis of Probe ***1***

#### 3.5.1. Compound **2**

The solution of compound **3** (524 mg, 2 mmol) containing K_2_CO_3_ (414 mg, 3 mmol) and phase transfer catalyst TBAHSO_4_ (2%) in DMSO (10 mL) was stirred at room temperature for 20 min. Propargyl bromide (80% in toluene) (476 mg, 4 mmol) was added drop-wise into the reaction mixture for 30 min and the resulting solution was stirred at room temperature for 48 h. The progress of the reaction was monitored by TLC (thin layer chromatography). After completion of the reaction, a brine solution was added into the reaction mixture, resulting in solid materials that were separated, filtered and dried. The obtained solid was purified by column chromatography to give a yellow product (compound **2**). Yield 70%, m.p. 90–92 °C; ^1^H NMR (CDCl_3_, 400 MHz): *δ* 2.26 (s, 1H, CH), 2.36 (s, 1H, CH), 3.85 (dd, *J*_1_ = 6.0 Hz, *J*_2_ = 1.2 Hz, 2H, CH_2_), 5.26 (s, 2H, CH_2_), 6.31 (t, *J* = 6.0 Hz, 1H, NH), 7.57 (t, *J* = 7.5 Hz, 1H, ArH), 7.67 (t, *J* = 7.8 Hz, 1H, ArH), 7.75–7.82 (m, 2H, 2 × ArH), 8.28 (d, *J* = 7.8 Hz, 1H, ArH), 8.36 (d, *J* = 7.8 Hz, 1H, ArH), 8.45 (d, *J* = 7.8 Hz, 1H, ArH); ^13^C NMR (CDCl_3_, 100 MHz): *δ* 33.06, 37.70, 42.03, 73.42, 77.27, 79.35, 111.67, 118.63, 118.79, 120.99, 123.37, 126.64, 127.53, 127.59, 128.33, 128.69, 131.64, 132.24, 132.75, 133.69, 137.21, 159.88, 182.11.

#### 3.5.2. Probe **1**

In a round-bottomed flask fitted with a septum, benzyl azide (532 mg, 4.0 mmol) was dissolved in a CH_2_Cl_2_-EtOH (1:1) mixture and degassed for 5 min with N_2_. Compound **2** (338 mg, 1.0 mmol) was added into the flask, with stirring under N_2_. Sodium ascorbate (6.0 mol%) was added, and reaction mixture was stirred for 5 min. Powdered CuSO_4_ (1.0 mol%) was added and stirring was continued until complete consumption of compound **2** (TLC). The solvent was removed under vacuum, and the resulting residue was partitioned between water and chloroform. The organic layer was collected and washed with water and brine solution. The organic solvent was removed after being dried over anhydrous Na_2_SO_4_. The resulting solid was purified by column chromatography to afford the product (probe **1**). Yield 74%, m.p. = 75 °C, HRMS (*m*/*z* + H^+^) = 605.2410, (theoretical (*m*/*z* + H^+^) = 605.2408); ^1^H NMR (CDCl_3_, 400 MHz): *δ* 4.31 (d, *J* = 6.0 Hz, 2H, CH_2_ of NHCH_2_), 5.40 (s, 2H, CH_2_), 5.44 (s, 2H, CH_2_), 5.68 (s, 2H, CH_2_), 6.59 (t, *J* = 6.0 Hz, 1H, NH), 7.11–7.14 (m, 2H, 2 × ArH), 7.22–7.34 (m, 9H, 9 × ArH), 7.52 (t, *J* = 7.5 Hz, 1H, ArH), 7.57 (d, *J* = 8.1 Hz, 1H, ArH), 7.61 (s, 2H, 2 × ArH), 7.72 (t, *J* = 7.5 Hz, 1H, ArH), 8.11 (d, *J* = 8.1 Hz, 1H, ArH), 8.30 (t, *J* = 7.7 Hz, 1H, ArH), 8.41 (d, *J* = 7.5 Hz, 1H, ArH); ^13^C NMR (CDCl_3_, 100 MHz): *δ* 39.91, 44.05, 54.48, 54.61, 111.09, 119.71, 121.56, 122.03, 123.73, 123.98, 126.96, 127.97, 128.30, 128.54, 128.80, 129.14, 129.19, 129.50, 132.01, 133.01, 134.47, 134.70, 134.80, 138.38, 143.71, 160.94, 183.40.

### 3.6. Live Cell Imaging

HeLa cells were grown in Minimum Essential Media (MEM) supplemented with 10% fetal bovine serum (FBS), 100 IU/mL penicillin and 100 µg/ml streptomycin. The cells were maintained in a humidified incubator at 37 °C with 5% CO_2_. A total of 3.8 × 10^4^ cells was seeded on a 13 mm glass bottom of a 35 mm confocal dish and grown for 24 hr (until 60–70% confluence). Experiments were performed in triplicate in FBS and antibiotic free medium as follows. HeLa cells were incubated with probe **1** (10 µM) at 37 °C with 5% CO_2_ for 30 min, followed by washing twice with 1 × phosphate-buffered saline (PBS) (pH = 7.4). The cells were further incubated with different concentrations of Cu^2+^ (0–20 µM) for 25 min under the same conditions. The cells were washed three times with 1 × PBS buffer. Confocal microscopy images were obtained from a K1-Fluo confocal laser scanning microscope. Imaging was performed using a 40× oil immersion objective lens. Bright-field images after treatment with probe **1** and Cu^2+^ were obtained to determine the viability of the cells under the conditions. 

## 4. Conclusions

We synthesized a new naphthoquinolinedione-based probe (probe **1**) containing two triazole units. The probe exhibited high selectivity for Cu^2+^ detection in 20% aqueous ethanol solution, and the probe-containing solution displayed a vivid color change from greenish-yellow to pink upon addition of the metal ion. A ratio-metric approach using two absorption values (***A***_438_ and ***A***_510_) corresponding to absorption maxima of the probe before and after Cu^2+^ addition, respectively, allowed us to detect Cu^2+^ as low as 0.5 µM. In addition, the green fluorescence emission was selectively quenched in the presence of Cu^2+^ among various metal ions. Hence, probe **1** provides dual detection of Cu^2+^ (absorption and fluorescence emission) and is chromo-fluorogenic. The effective fluorescence of the probe appeared to occur mainly via a dynamic process, as supported by the significantly reduced excited state life-time of the probe in the presence of the metal ion. The fluorescence titration study of probe **1** gave a very low calculated LOD (41.5 pM). UV-vis and fluorescence titrations as well as DFT calculations support the 1:1 complex formation between probe **1** and Cu^2+^. When the probe was used to sense Cu^2+^ dissolved in real samples such as tap and lake water, the probe LOD did not change much, indicating its potential in on-site monitoring of the metal ion. Additionally, the probe effectively penetrated cell membranes to detect Cu^2+^ in HeLa cells. This study gives insight into the development of new chromo-fluorogenic heteroaromatic sensors for detection of different analytes.

## Data Availability

Data supporting reported results can be found in the electronic supplementary information (ESI).

## Supplementary Materials

The following are available online, Figure S1: Effects of different metal ions on UV-Vis and fluorescence spectrum of probe 1, Figure S2: Effect of solvent composition on fluorescence spectrum of probe 1 in the presence of Cu2+, Figure S3: Interference study of various metal ions on Cu2+ sensing of probe 1, Figure S4: Linear plot between the fluorescence intensity of probe 1 and [Cu2+], Figure S5: Energy-minimized structures of probe 1 and its Cu2+ complex obtained from DFT calculations, Figure S6: Stern-Volmer plot of probe 1 as a function of [Cu2+], Figure S7: Co-localization images of probe 1 in HeLa cells, Table S1: LOD comparison of probe 1 with other probes previous reported for Cu2+ detection.

## Sample Availability

Sample of the compound (probe **1**) is available from the authors.
