# Supplementary material for: A Chromo-Fluorogenic Naphthoquinolinedione-Based Probe for Dual Detection of Cu2+ and Its Use for Various Water Samples
Source: Molecules. 2022 Jan 25;27(3):785. doi: 10.3390/molecules27030785 (PMC8838320; doi:10.3390/molecules27030785)
Supplement: Supplementary file 1 [file molecules-27-00785-s001.zip › molecules-1552313-supplementary.pdf]

ESI

A chromo-fluorogenic naphthoquinolinedione-based probe for dual detection of Cu^{2+} and its use for various water samples

Ashwani Kumar,[1,*] Subodh Kumar[b], Pil Seok Chae,[a,*]

[1] Department of Bionano Engineering, Hanyang University, Ansan, 155-88, Republic of Korea,
[2] Department of Chemistry, UGC Center for Advanced Studies, Guru Nanak Dev University, Amritsar-143005, Punjab, India

* Correspondence: ashwanirubal@gmail.com, pchae@hanyang.ac.kr

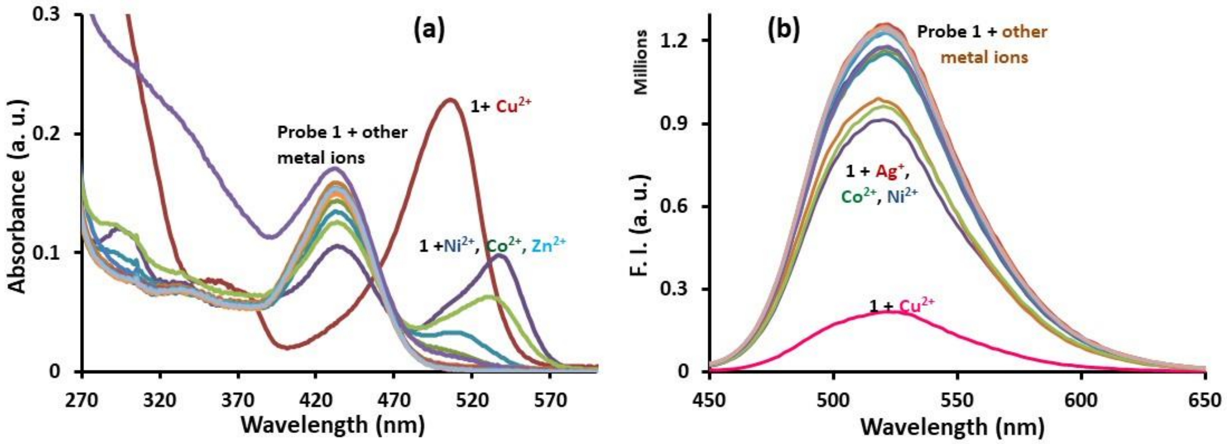

Figure S1. Effects of different metal ions on (a) UV-Vis and (b) fluorescence spectrum of probe **1** (10 µM, EtOH). Tested ions include monovalent (Na^{+} and K^{+}), divalent (Mg^{2+}, Ca^{2+}, Ba^{2+}, Fe^{2+}, Co^{2+}, Zn^{2+}, Ag^{+}, Cd^{2+}, Pb^{2+}, Cu^{2+}, Ni^{2+}, and Hg^{2+}), and trivalent metal ion (Cr^{3+}, Fe^{3+}, Ga^{3+}, Al^{3+}, and Ru^{3+}).

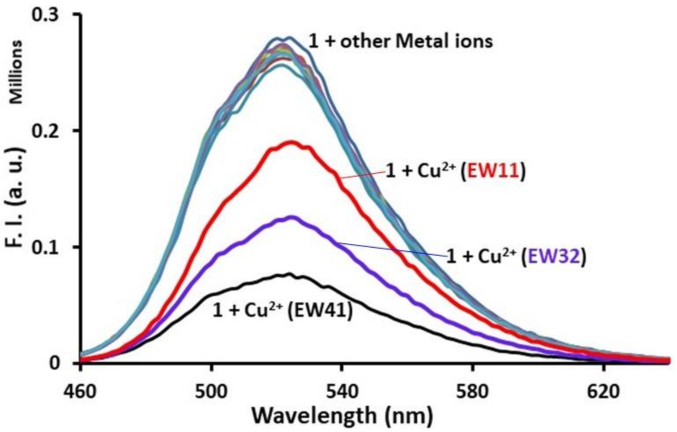

Figure S2. Effect of solvent composition on fluorescence spectrum of probe **1** (10 µM, EtOH) in the presence of Cu^{2+}; λ_{ex} = 435 nm. Solvent composition varied from 20% (EW41) to 40% (EW32) to 50% (EW11). 'E' and 'W' stand for ethanol and water, respectively. The numbers indicate the ratios of ethanol and water.

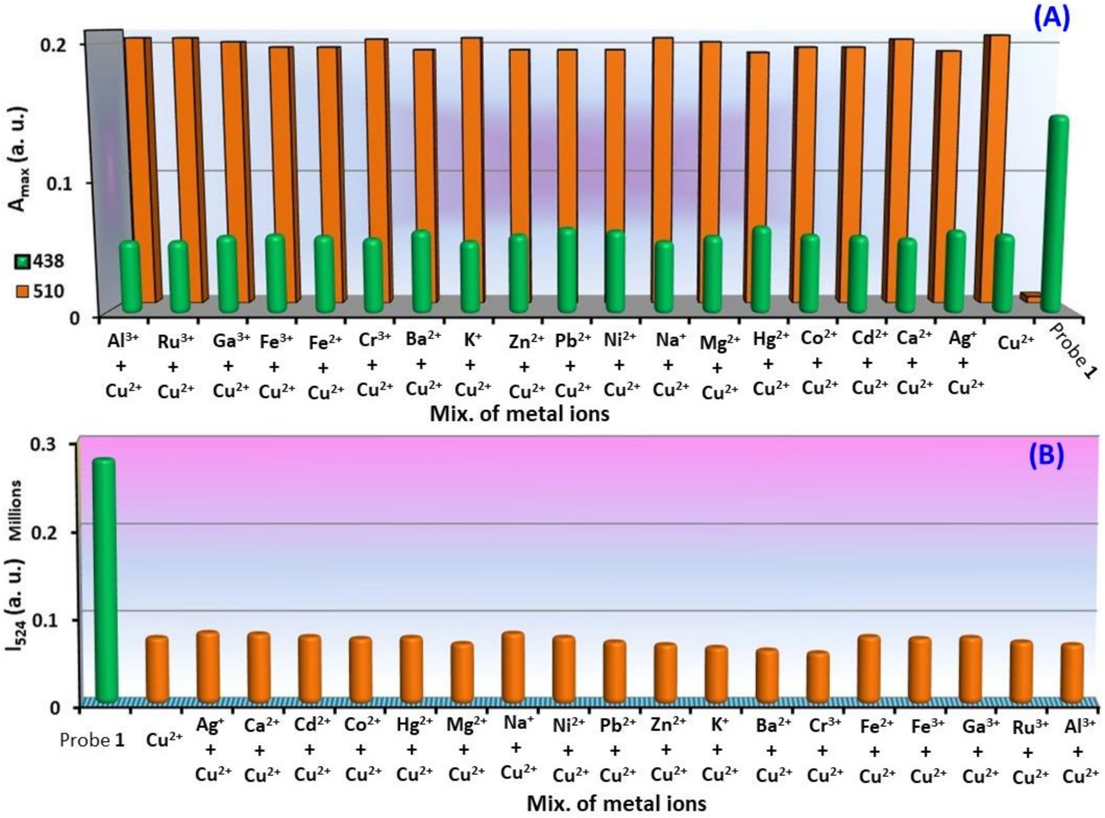

Figure S3. Interference study of variou smetal ions (200 µM each) on Cu^{2+} sensing of probe **1** (10 µM, HEPES (pH 7.4)-EtOH (1:4)). (A) Bar diagram of the absorbance at 438 nm (green) and 510 nm (orange), and (B) fluorescence intensity of probe **1** at 524nm (I_{524}) in the presence of Cu^{2+} upon additions of individual metal ions.

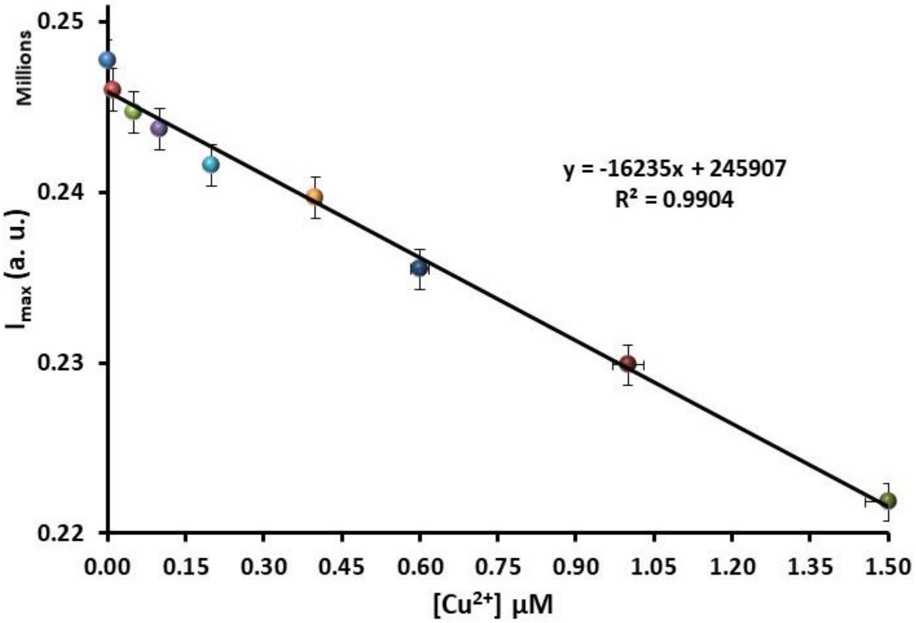

Figure S4. Linear plot between the fluorescence intensity of probe **1** (10 µM, HEPES (pH 7.4): EtOH =1:4) and [Cu^{2+}]. The probe was excited at 435 nm (λ_{ex}) and the resulting fluorescence intensity was measured at 524 nm ($\boldsymbol{I}_{524}$).

LOD calculation of probe 1 for Cu^{2+} detection

A probe LOD was calculated from the fluorescence titration data. The slope (*K*) was obtained from the linear plot of the maximum fluorescence intensity of the probe ($\boldsymbol{I}_{524}$) and [Cu^{2+}]. The fluorescence emission spectra of the probe were measured three times, and the standard deviation (σ) of the blank measurement was obtained.

LOD = 3 x σ/K

σ = Standard deviation of blank measurement = 0.22435

K = Slope of the linear plot between the maximum fluorescence intensity of the probe ($\boldsymbol{I}_{524}$) vs [Cu^{2+}] = -16235 x 10^6

Detection Limit (LOD) = 3 x 0.22435/16235 x 10^6 = 4.145 x 10^{-11} M = 41.5 *pM*

Table S1. LOD comparison of probe **1** with other probes previous reported for Cu^{2+} detection.

S. No.	Probe structure	Detection limit in solution (LOD(Flu.))	Solution, selectivity	Reference
**1.**	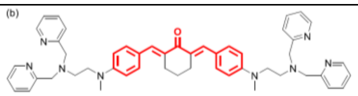	0.379 μM	MeOH/HEPES, pH 7 (1:1)<br>Cu^{2+} selective<br>Respond to Ni^{2+}, Co^{2+}, Zn^{2+}, Cd^{2+}	Dyes Pigments 197 (2022) 109845
**2.**	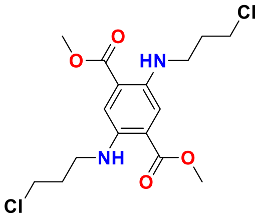	0.303 μM	acetonitrile-water (v/v = 9:1)<br>Cu^{2+} and<br>TFA & TEA	Dyes Pigments 197 (2022) 109903
**3.**	carbon dots (h-CDs)	1.8×10^{-4} mmol	water	Dyes Pigments 198 (2022) 110008
**4.**	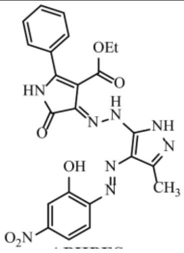	0.59–0.98 μM	EtOH:Tris HCl buffer (9:1)<br>Cu^{2+}, Zn^{2+}, Co^{2+}, only colorimetric	Dyes Pigments 196 (2021) 109795
**5.**	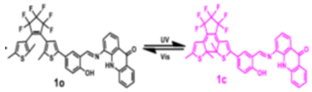	0.395 μM	DMSO<br>Arginine, Cu^{2+}<br>only colorimetric	Dyes Pigments 195 (2021) 109752
**6.**	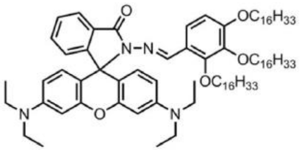	0.829 nM.	Cyclohexanol	Dyes Pigments 192 (2021) 109436

**7.**	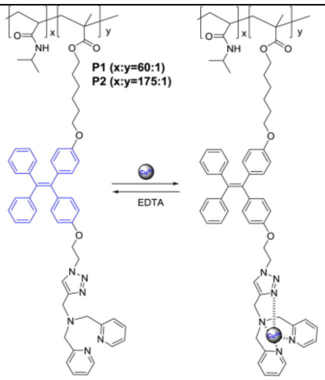	57 nM	MOPS buffer solution	Sensors and Actuators: B. Chemical 344 (2021) 130241
**8.**	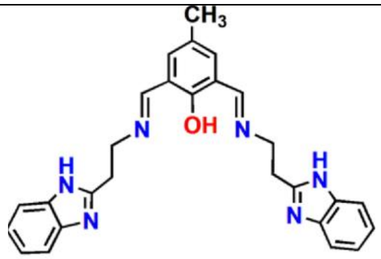	24.4 nM	CH_3CN:HEPES (1:1)<br>Cu^{2+}, Zn^{2+}, CN^-, $P_2O_7^{4-}$, DNA, sensing	Sensors & Actuators: B. Chemical 337 (2021) 129785
**9.**	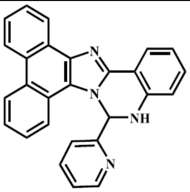 PIQPy	0.1 µM	water/EtOH (1:1, HEPES) pH 7.4	Dyes Pigments 173 (2020) 107916
**10.**	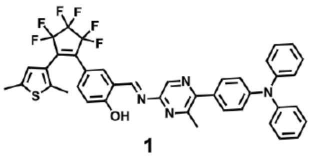 1	2.0×10^{-6} M	THF	Dye Pigments 173 (2020) 107914.
**11.**	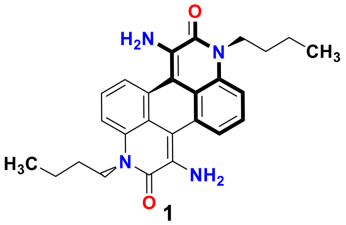 1	5 nM	(HEPES:CH_3CN (1:1), pH 7.4)	Dyes and Pigments 181 (2020) 108522

**12.**	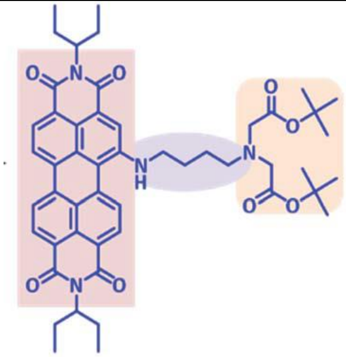	4.8 μM	HEPES: CH_3CN (4: 6, pH 7.2)	**Anal. Methods**, 2020,**12**, 758-767
**13.**	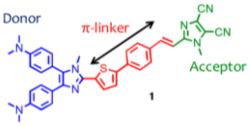	0.53×10^{-6} M	acetonitrile−water (1:1)	J. Org. Chem. 84 (2019) 1787-1796
**14.**	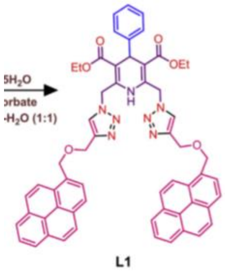	0.53×10^{-6} M	CH_3CN	Dyes and Pigments 161 (2019) 162–171
**15.**	**ZnS** microhybrid particles constructed from interfacial coordination bridge of thiocyanate	1×10^{-6} M	THT	Dyes and Pigments 172 (2020) 107831
**16.**	Sponge cellulose fluorescence spherical (CS—CDs)	0.11 μM	water	ACS Sustainable Chem. Eng. 7 (2019) 19157–19166
**17.**	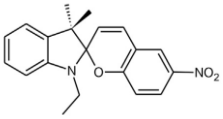	10^{-12} M	DMF/H_2O (9/1, v/v)	Anal. Chim. Acta 1061 (2019) 161-168
**18.**	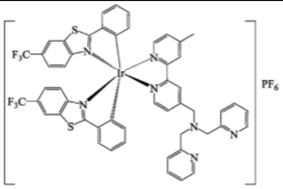<br>$[(CF_3\text{-bt})_2Ir(bp\text{-DPA})]PF_6$	0.5×10^{-6} M	CH_3OH	Dyes Pigments 161 (2019) 252–260

**19.**	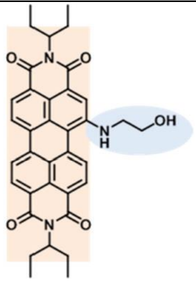	3 nM	HEPES: CH_3CN (1:1) pH 7.2	*J. Mater. Chem. B,* 7 (2019) 7218-7227
**20.**	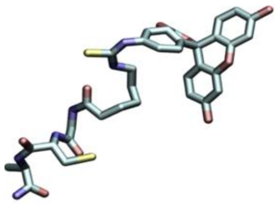 L (FITC-Ahx-GCA-NH_2)	37 nM	HEPES pH 7.4	Sens. Actuators B 280 (2019) 129–137
**21.**	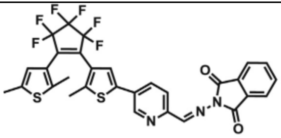	4.12×10^{-8} M	methanol	Dyes Pigments 161 (2019) 34–43
**22.**	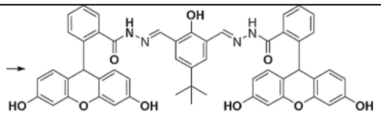	0.21 μM	ACN-H_2O (3:7) pH 7.0 (HEPES)	Sens. Actuators B 282 (2019) 730–742
**23.**	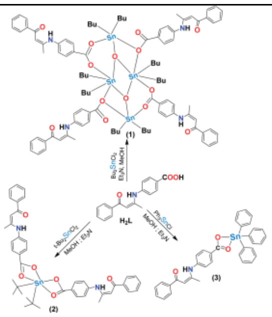	1.47 μM	methanol	New J. Chem. 43 (2019) 16050--16057
**24.**	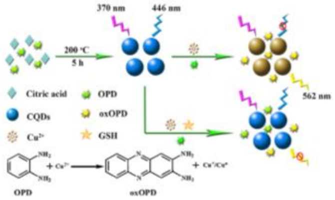	0.25 μM	Tris-HCl buffer (pH 7.4, 50 mmol L^{-1}).	Sens. Actuators B 298 (2019) 126842

**25.**	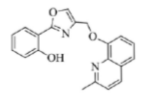 L1 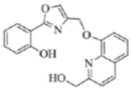 L3	8.24 × 10^{-8} M	DMF-H2O (1:1) Tris-HCl buffer pH 7.3	Dyes Pigments 161 (2019) 331-340.
**26.**	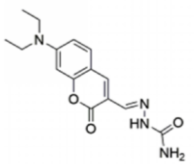	2×10^{-8} M	EtOH-H_2O (1:1)	Sens. Actuators B 288 (2019) 27–37
**27.**	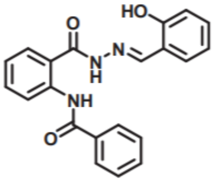	2.26 × 10^{-6} M	CH_3OH-H_2O (1:1)	Sensors & Actuators: B. Chemical 275 (2018) 350–358
**28.**	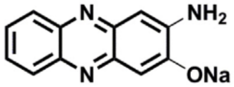	2.2×10^{-7} M	water	Dyes Pigments 171 (2019) 107707
**29.**	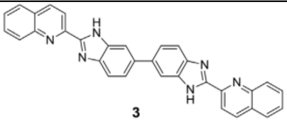 3	1.12 nM (calc.) 0.05 µM (obs.)	DMSO/H_2O, (9/1)	Dyes Pigments 170 (2019) 107651
**30.**	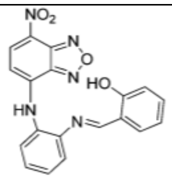	1.8 x 10^{-7} M	(CH_3OH: H_2O) 1:1, HEPES	New J. Chem. 40 (2016) 2418--2422
**31.**	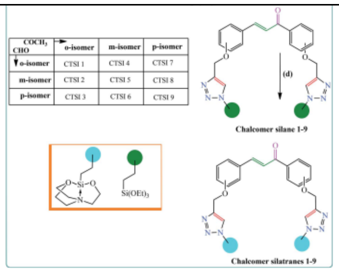	10 × 10^{-6} M	MeOH: H_2O (8: 2)	RSC Adv. 5 (2015) 12644–12654
**32.**	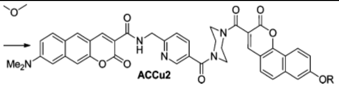	0.84 × 10^{-6} M	EtOH/HEPES (9/1, v/v, pH 7.0)	Anal. Chem. 86 (2014) 5353—5359

**33.**	**pytz capped AgNPs**<br>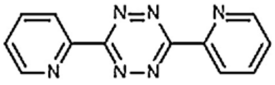<br>Pytz	17.5×10^{-6} M ,<br>9.4×10^{-6} M,<br>13.2×10^{-6} M,<br>Ni^{2+}, Ag^{+},<br>respectively	aqueous solution	Sens. Actuators B 202 (2014) 23–30
**34.**	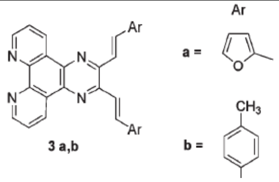	3×10^{-6} M	MeOH	Dalton Trans., 2012, 41, 5769–5773
**35.**	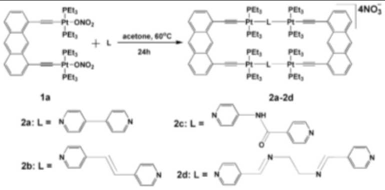	7.33×10^{-4} M	MeOH	Inorg. Chem. 2009, 48, 549-556.
**36.**	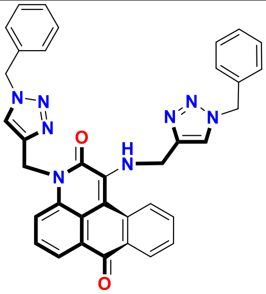<br>**1**	**41.5 *pM (calc.)***	[HEPES (pH 7.4): EtOH = 1:4)]	***Present work***

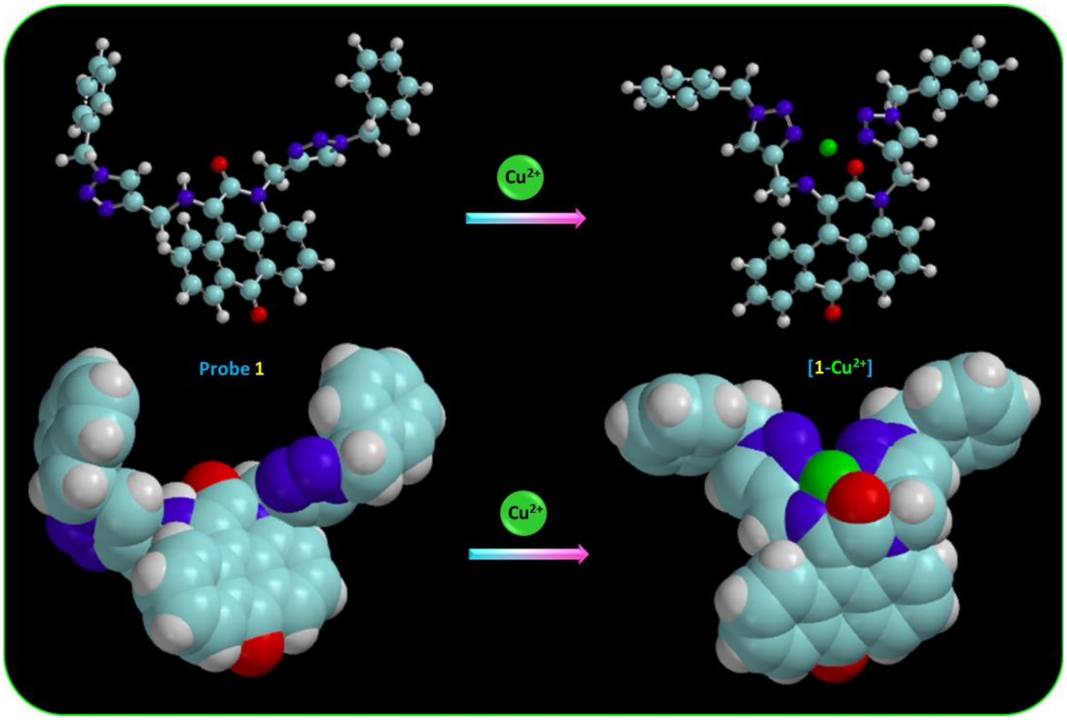

Figure S5. Energy-minimized structures of probe **1** and its Cu^{2+} complex obtained from DFT calculations at the B3LYP/6-31G* level. (*Top*) Ball-and-stick model and (*bottom*) space-filling model. Color coding: C = cyan, N = blue, O = red, Cu^{2+} = green, and H = white.

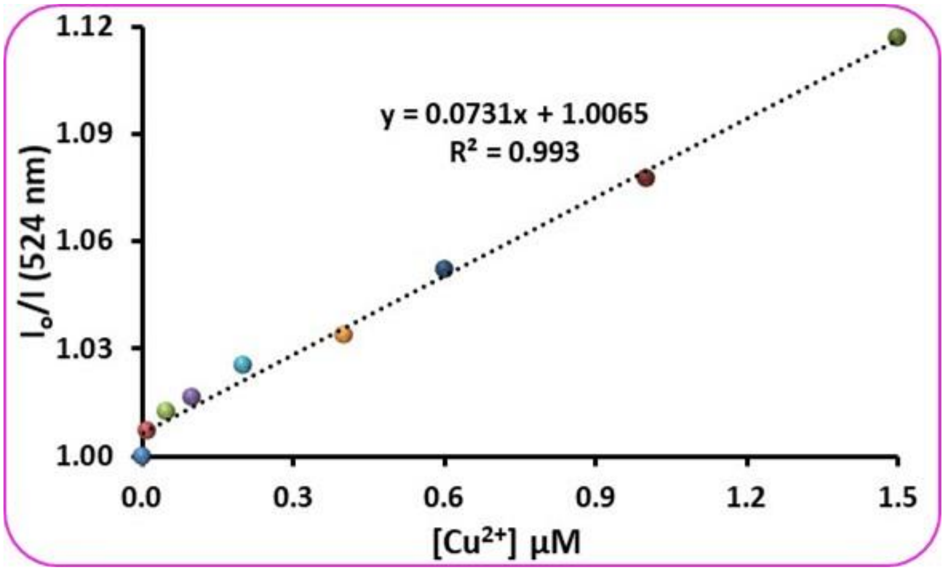

Figure S6. Stern-Volmer plot of probe **1** (10 μM, HEPES (pH 7.4): EtOH =1:4) as a function of [Cu^{2+}]. $\boldsymbol{I_o}$ and $\boldsymbol{I}$ represent fluorescent emission intensities of the probe in the absence and presence of Cu^{2+}, respectively.

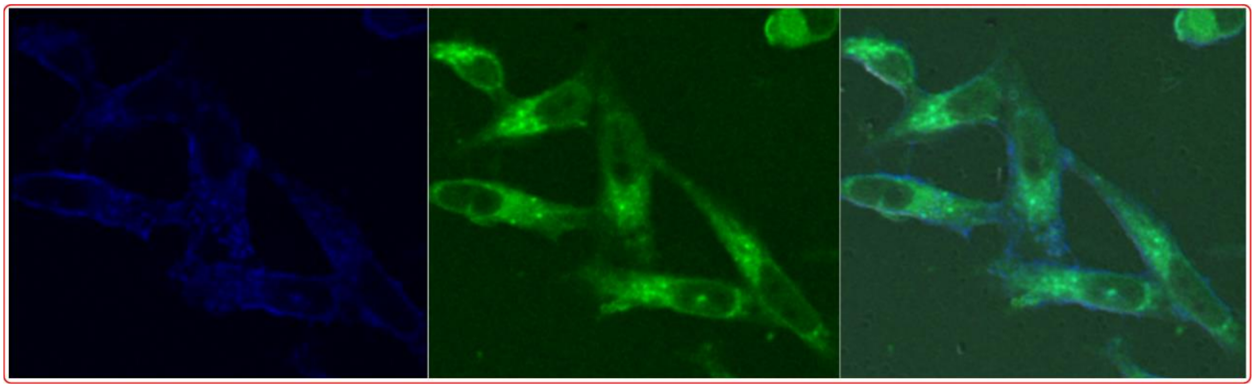

Figure S7. Co-localization images of probe **1** in HeLa cells. The cells were incubated with the probe for 30 min. (*left*) Blue channel; (*center*) green channel; (*right*) overlap of two in dark field. The probe is localized within cytoplasm in a high density, while the probe density was very low in the nucleus.

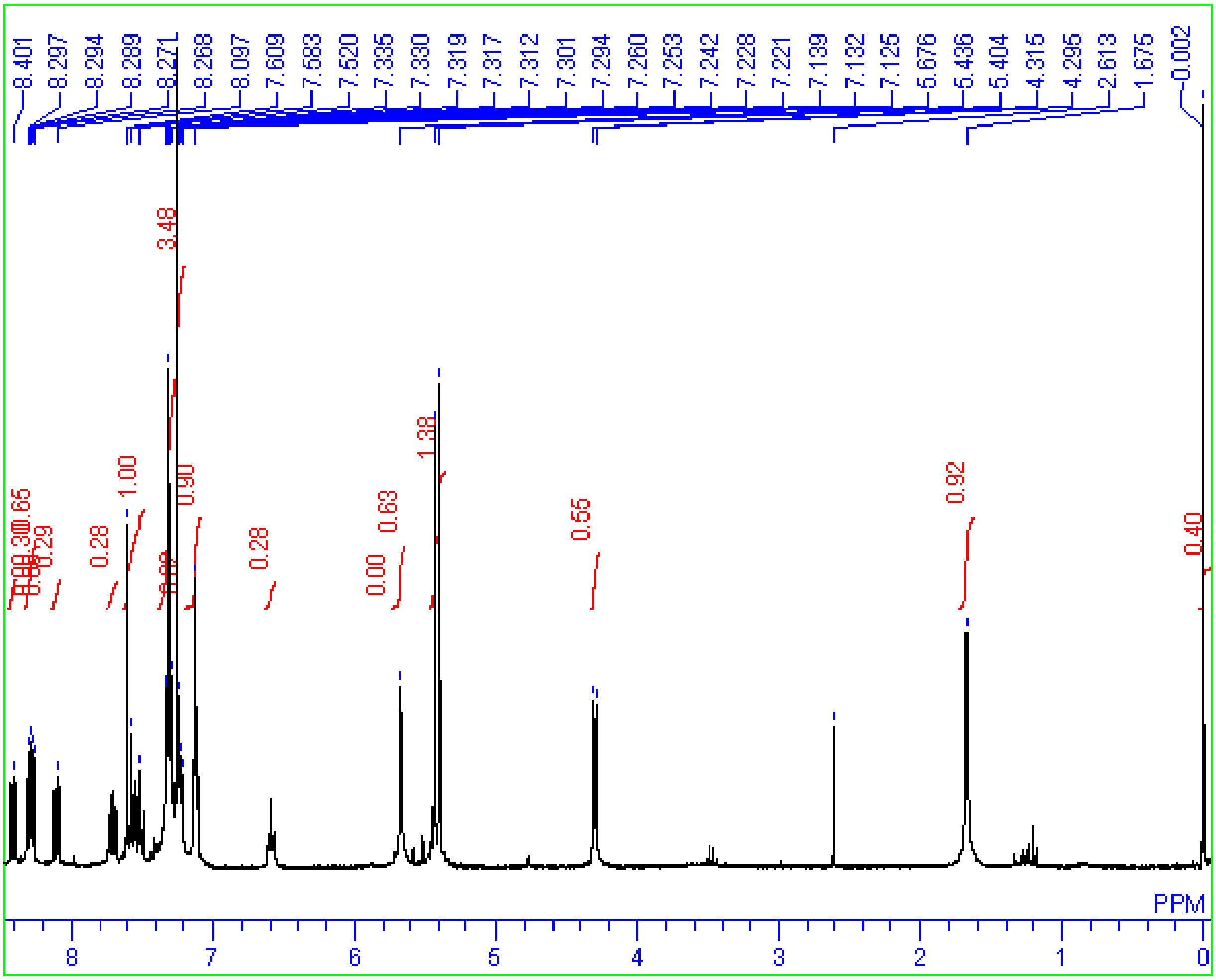

^{1}H NMR spectrum of probe **1** in $CDCl_3$

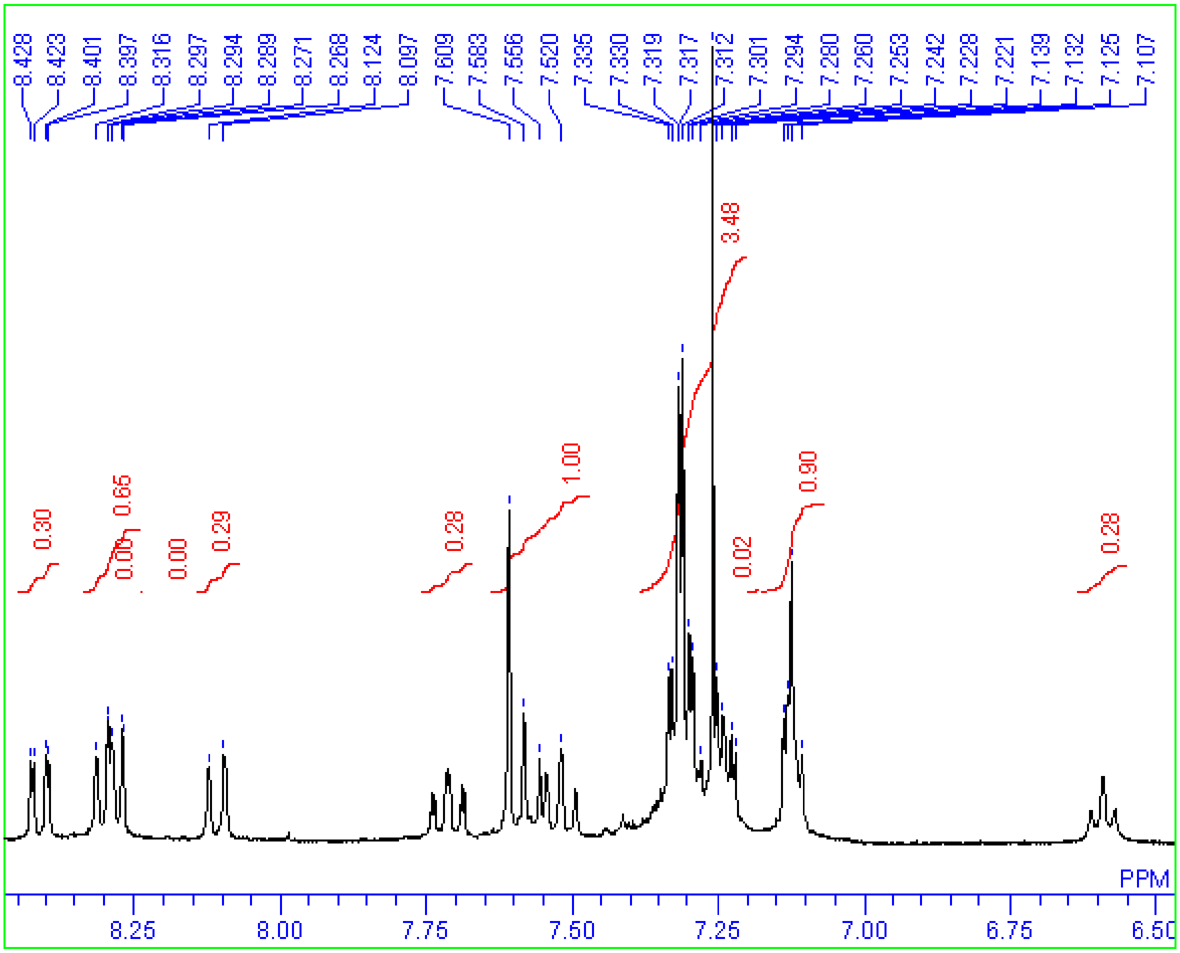

^1H NMR spectrum of probe **1** (aromatic region) in $CDCl_3$

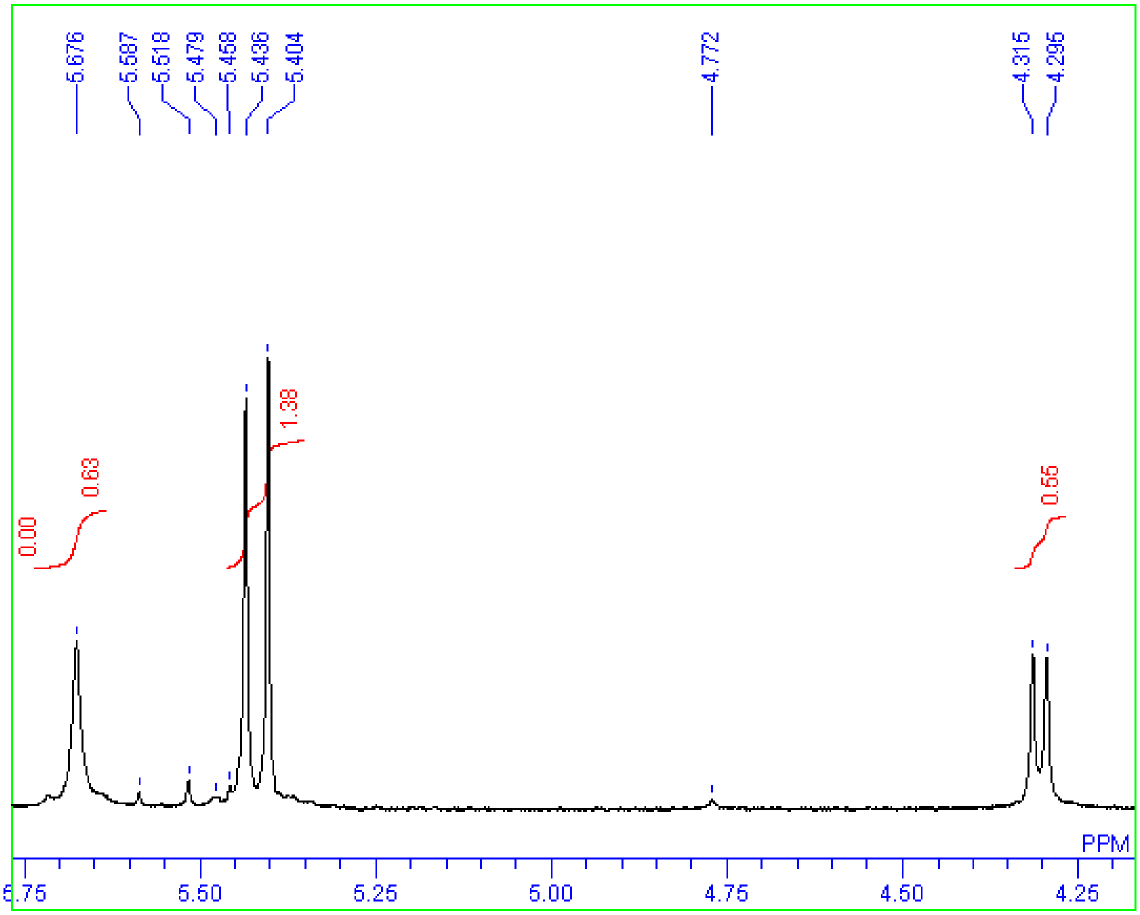

^{1}H NMR spectrum of probe **1** (aliphatic region) in $CDCl_3$

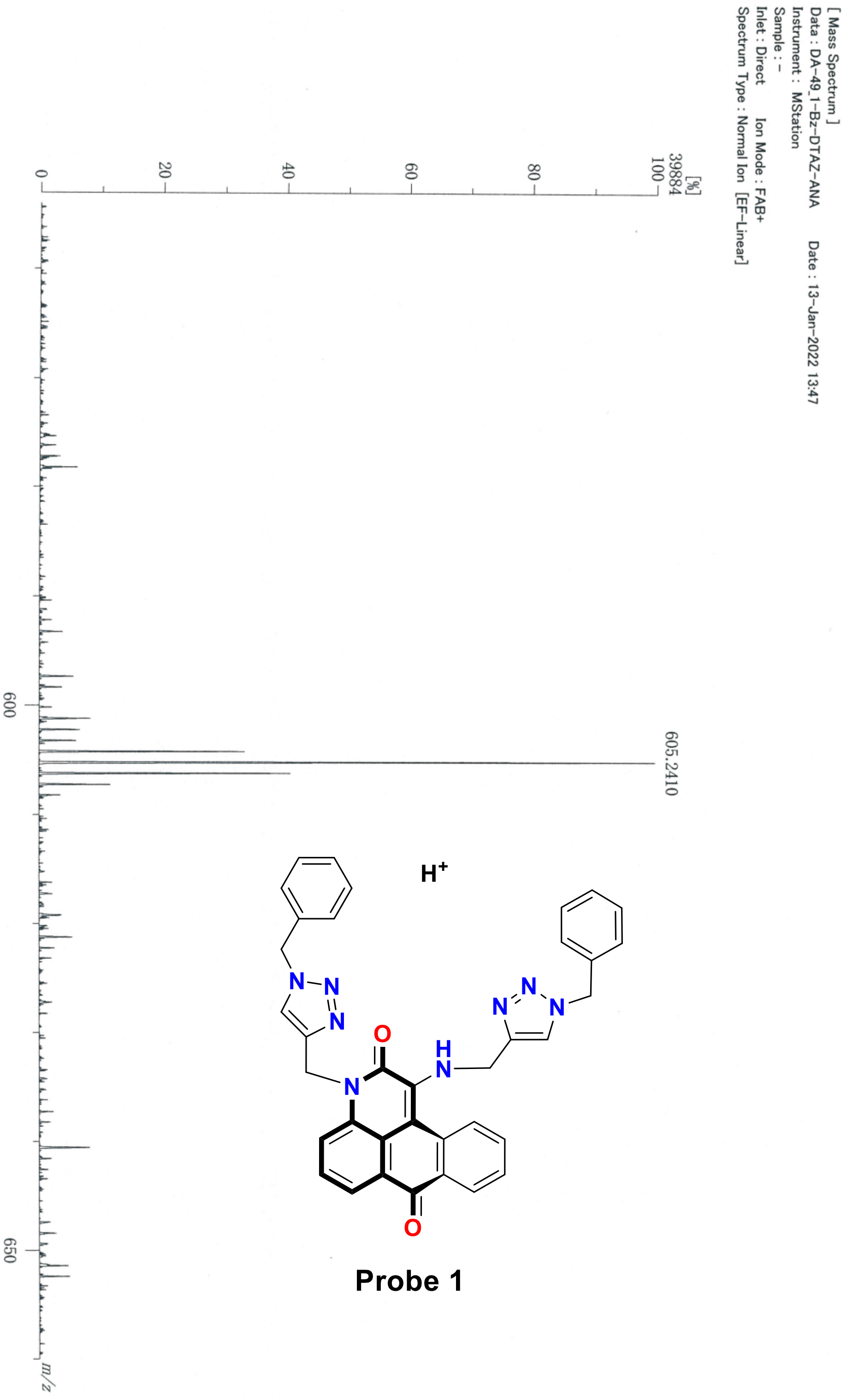

HRMS of probe **1**

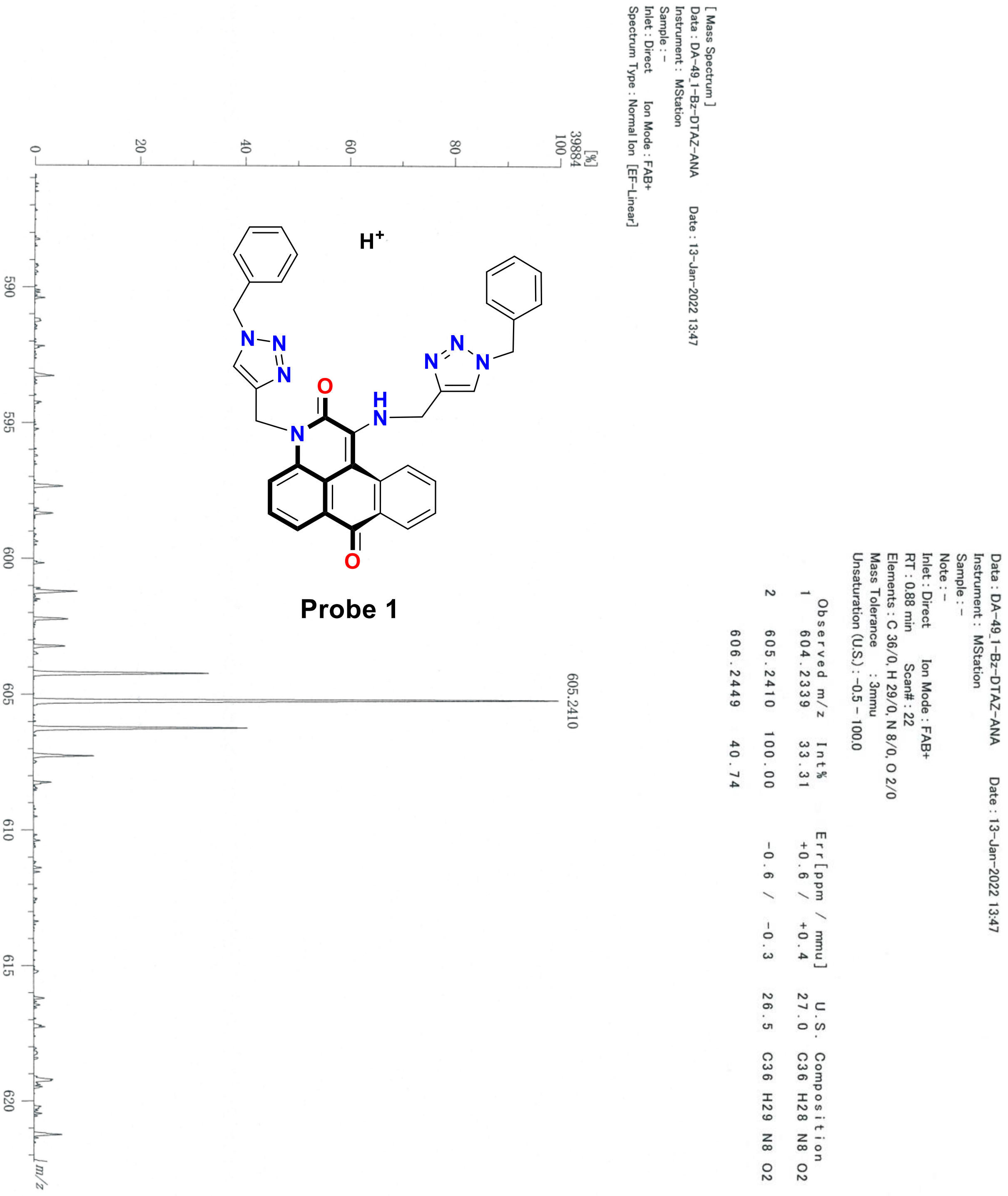

HRMS of probe **1**

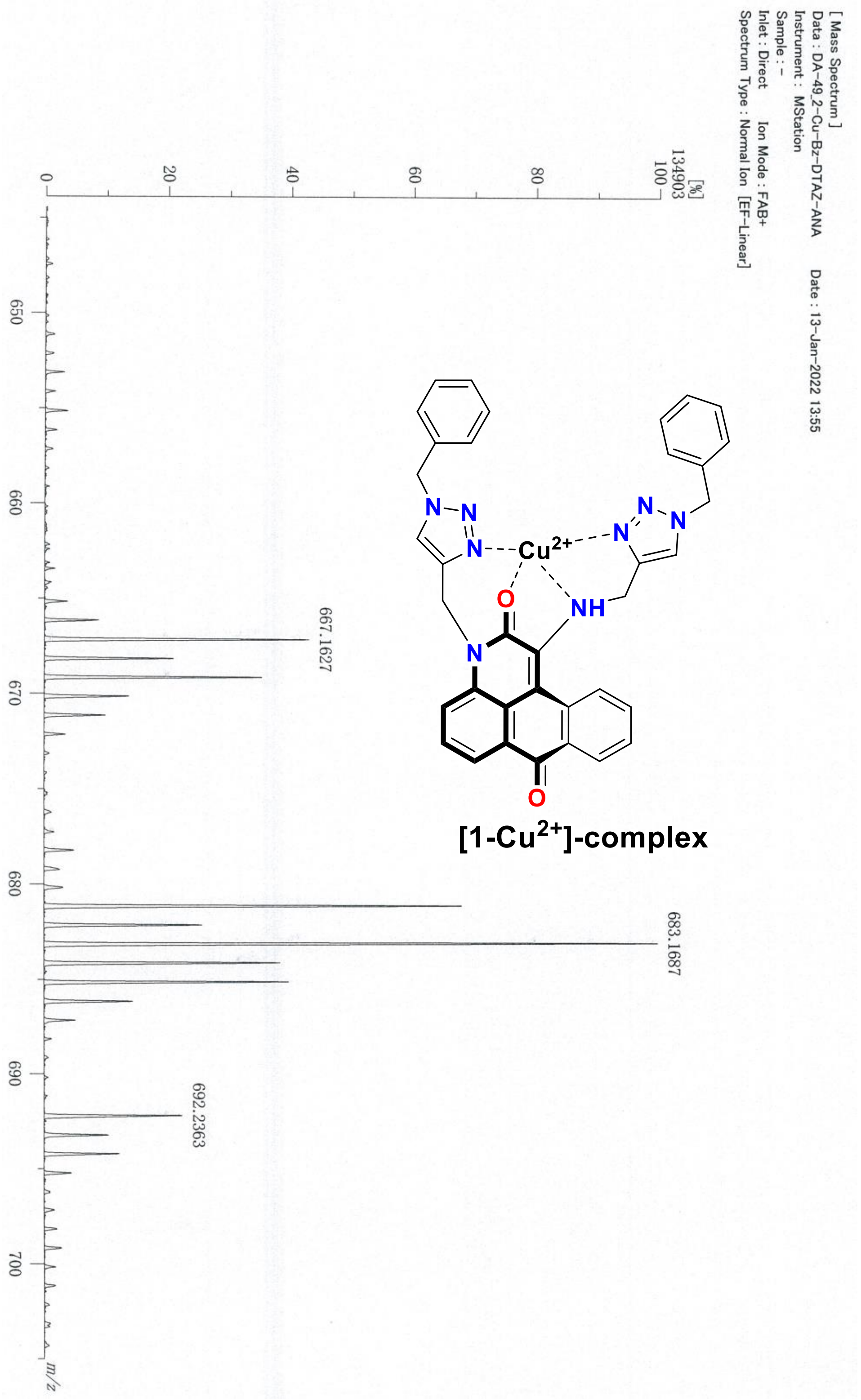

HRMS of [**1-Cu**$^{2+}$]-complex

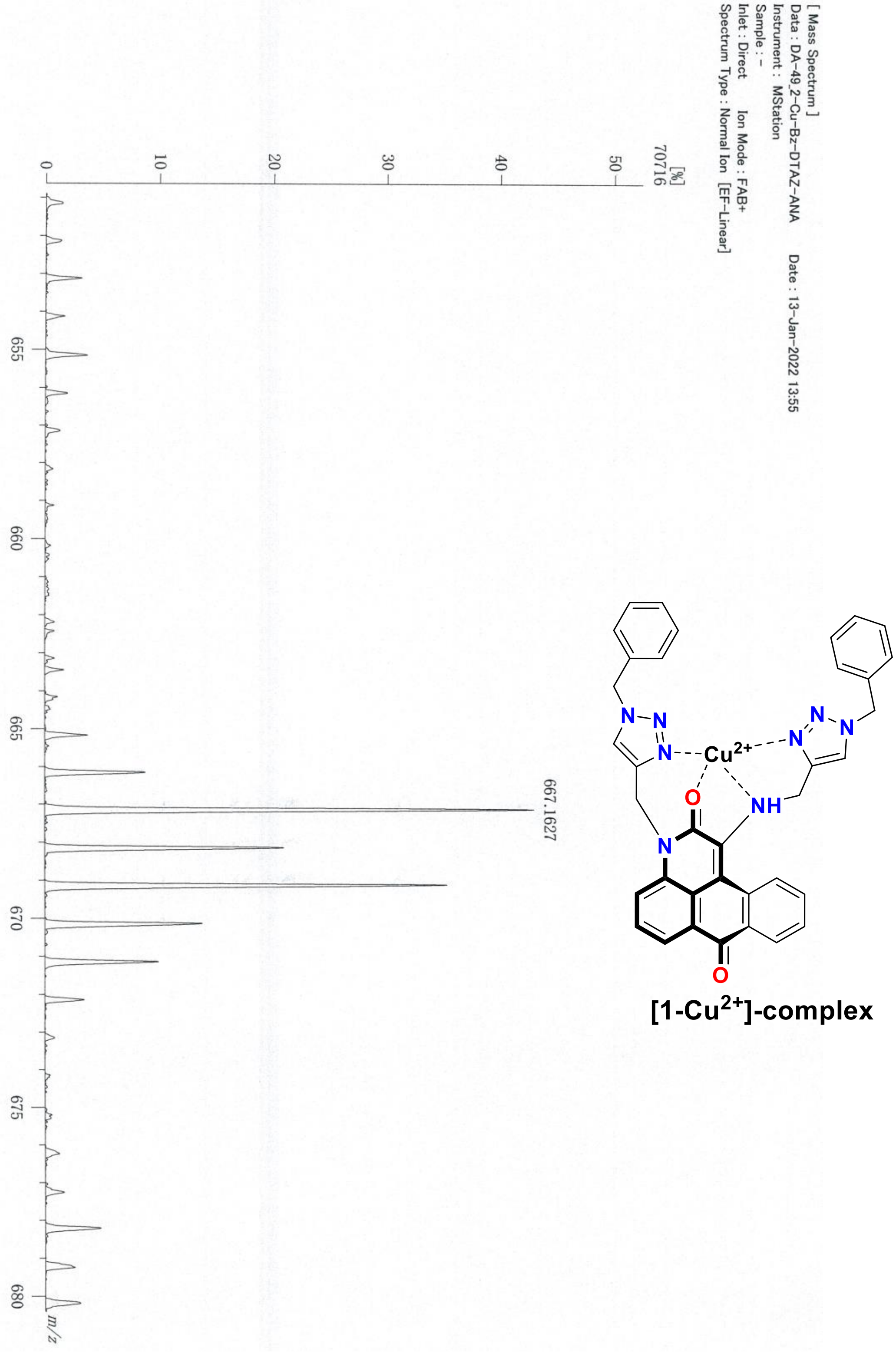

HRMS of [**1-Cu^{2+}**]-complex
